# The Cambrian cirratuliform *Iotuba* denotes an early annelid radiation

**DOI:** 10.1098/rspb.2022.2014

**Published:** 2023-02-08

**Authors:** ZhiFei Zhang, Martin R. Smith, XinYi Ren

**Affiliations:** ^1^ State Key Laboratory of Continental Dynamics, Shaanxi Key Laboratory of Early Life and Environments and Department of Geology, Northwest University, Xi'an 710069, People's Republic of China; ^2^ Department of Earth Sciences, Durham University, Mountjoy Site, South Road, Durham DH1 3LE, UK

**Keywords:** Cambrian explosion, annelid evolution, body plans, phylogenetics, polychaetes

## Abstract

The principal animal lineages (phyla) diverged in the Cambrian, but most diversity at lower taxonomic ranks arose more gradually over the subsequent 500 Myr. Annelid worms seem to exemplify this pattern, based on molecular analyses and the fossil record: Cambrian Burgess Shale-type deposits host a single, early-diverging crown-group annelid alongside a morphologically and taxonomically conservative stem group; the polychaete sub-classes diverge in the Ordovician; and many orders and families are first documented in Carboniferous Lagerstätten. Fifteen new fossils of the ‘phoronid’ *Iotuba (=Eophoronis) chengjiangensis* from the early Cambrian Chengjiang Lagerstätte challenge this picture. A chaetal cephalic cage surrounds a retractile head with branchial plates, affiliating *Iotuba* with the derived polychaete families ‘Flabelligeridae’ and Acrocirridae. Unless this similarity represents profound convergent evolution, this relationship would pull back the origin of the nested crown groups of Cirratuliformia, Sedentaria and Pleistoannelida by tens of millions of years—indicating a dramatic unseen origin of modern annelid diversity in the heat of the Cambrian ‘explosion’.

## 1. Introduction

Annelids are a taxonomically and morphologically diverse animal phylum with deep evolutionary origins [1]. As most annelid lineages lack the recalcitrant hard parts necessary for preservation by conventional fossilization processes, the patterns and timing of their diversification must be inferred from a sparse fossil record [2]. The earliest unequivocal annelids occur in Burgess Shale-type Cambrian Lagerstätten and predominantly belong to the stem group [3,4], with a single representative of the crown group (*Dannychaeta*) from the early-diverging magelonid lineage [5]. The available fossil record denotes an accumulation of class-level diversity during the Ordovician Biodiversification Event [3,6], with many orders and families represented in the Carboniferous [2]—an overall trend that broadly aligns with the results of molecular analyses [7]. Nevertheless, the depauperate nature of the annelid fossil record means even a single fossil find can prompt significant revisions of evolutionary history [5].

Burgess Shale-type fossils are particularly relevant to annelid origins: their early-to-mid Cambrian age potentially illuminates the earliest stages of the diversification of the group, and their unrivalled preservation of fine-scale microstructural and anatomical detail allows the reconstruction of soft tissues that would never otherwise be preserved [8,9]. This said, extreme compression [10] and complicated preservational pathways [11] can complicate fossil interpretation: Burgess Shale ‘annelids’ [9,12] have later been reassigned to phyla as different as Onychophora [13], Priapulida [14] and Mollusca [15]. Here, we identify a likely mis-interpretation in the opposite direction: 15 new specimens of the Chengjiang [16] fossil *Iotuba chengjiangensis*, originally interpreted as tentaculate stalked phoronids with U-shaped guts [17,18], instead exhibit features of flabelligeroid annelids.

## 2. Results

**Clade:** Pleistoannelida Struck 2011 [19].

**Subclass:** Sedentaria Lamarck 1818 [20].

**Suborder:** Cirratuliformia Rouse & Pleijel 2003 [21].

Cirratuliformia has traditionally been included in the order Terebellida, which is no longer held to be monophyletic [22].

**New superfamily:** Flabelligeroidea.

*Type genus. Flabelligera* de Saint-Joseph, 1894 [23].

In view of uncertainty as to whether ‘Flabelligeridae’ represents a sister clade to [24,25], or a grade embracing [26], Acrocirridae, it is convenient to name the clade comprising ‘Flabelligeridae’ and Acrocirridae, which is consistently recovered by phylogenetic analyses.

*Iotuba* Zhang *et* Smith gen. nov.

*Type species. Iotuba chengjiangensis* sp. nov., by monotypy.

*Diagnosis*. As for the type species, by monotypy.

*Iotuba chengjiangensis* Zhang et Smith gen. *et* sp. nov.

*Remarks.* The names *Iotuba chengjiangensis* Chen *et* Zhou 1997 [17], its misspelling *Lotuba* [27], and *Eophoronis chengjiangensis* Chen 2004 [18, p. 216] are *nomina nuda* under article 13.1 of the International Code of Zoological Nomenclature [28], as they have never been accompanied by a formal diagnosis. We thus formally establish *Iotuba chengjiangensis* gen. *et* sp. nov. herein.

*Holotype.* Early Life Research Centre, Yunnan 53001 is designated as the holotype, following the intention of previous researchers [17,18].

*Additional material.* Complete specimen: Early Life Institute (ELI) S-001; anterior trunk (12 specimens): ELI S-002–008, S-010, S-011, S-014–016; medial trunk (two specimens, each questionably assigned to the species): ELI S-012, S-013; posterior trunk (one specimen): ELI S-009.

*Provenance.* Yellowish-green to greyish-green mudstones in the Jianshan, Ercaicun, Erjie and Sanjiezi sections of the middle-upper Yu'anshan Formation, *Eoredlichia* trilobite Zone, Cambrian Series 2, Stage 3, near Haikou, Kunming, Yunnan. Fossils were deposited in shallow waters with a freshwater, potentially deltaic, influence [29,30], and are preserved in the characteristic Chengjiang fashion [31] as weathered carbonaceous films associated with superficial iron oxides.

*Diagnosis.* Worms with subcylindrical trunk and eversible anterior region (head). Head slightly longer than wide when fully everted, bearing irregularly distributed conical papillae and two peripherally digitate horseshoe-shaped structures. Anteriormost trunk with palisades and fascicles of elongate spines. Trunk bearing transverse rows of small (*ca* 200 µm) conical papillae. Straight digestive tract flanked by pair of elongate tubes.

**Figure 1.**
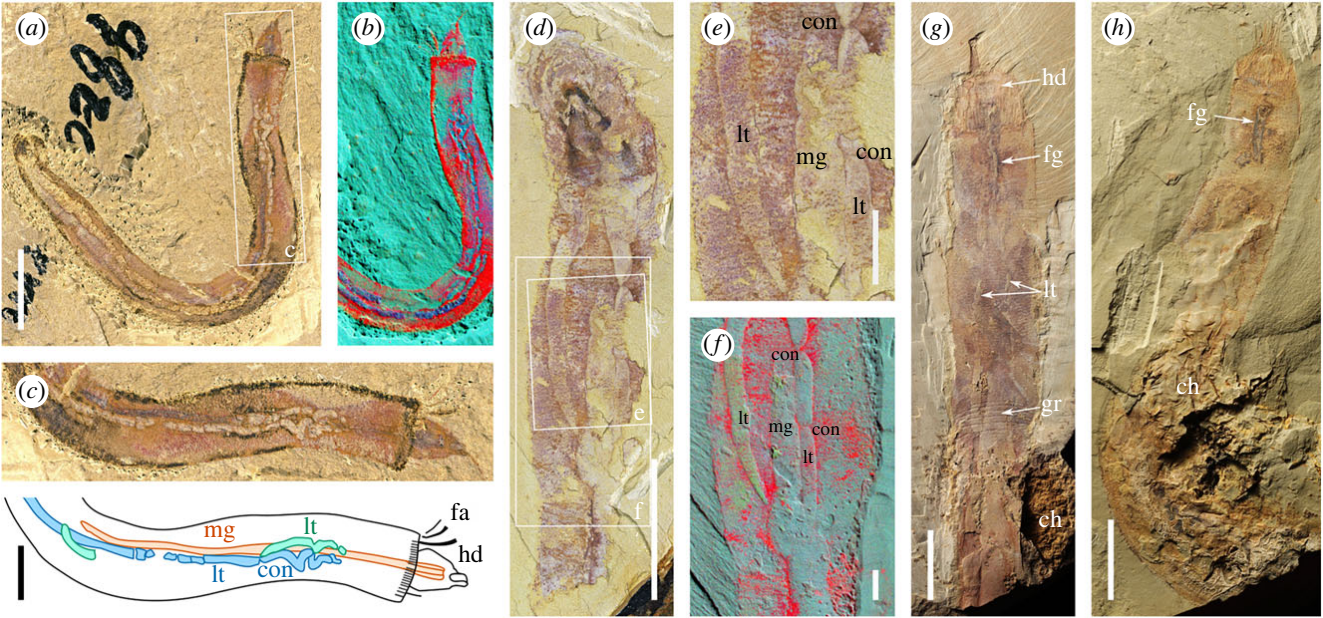
*Iotuba chengjiangensis*. (*a–c*) ELI-S-001, complete specimen with recurved gut and head partly retracted; (*b*) normalized elemental abundance measured by micro-X-ray fluorescence: blue channel, Al + K; green, Si + Zn + N; red, P + Fe; (*d–f*) ELI-S-002A, anterior trunk with boudinaged gut; head preserved perpendicular to plane of splitting; blue channel in (*f*): normalized abundance of Al + K; green, Si; red, P + Fe + Cr + Cu; (*g*) ELI-S-007A, anterior end everted; chancelloriid associated with posterior trunk; the iron-rich region anterior to the head is on a different surface and is not part of the *Iotuba* fossil; (*h*) ELI-S-003B, chancelloriid associated with posterior trunk. High-resolution images at *Figshare* [32]. Scale bars: 10 mm except enlargements (*c,e–f*), 2 mm. Abbreviations: ch, chancelloriid; con, constriction between boudins; fa, fascicle of spines; fg, foregut; gr, transverse groove; hd, everted head; lt, lateral tube; mg, midgut.

*Description.* Specimens range from 3.2 to 12.1 mm in width (figure 1); the aspect ratio of the single complete specimen (figure 1*a–c*) is 10.6. Individuals are often bent close to their mid-trunk, by 5–170°.

**Figure 2.**
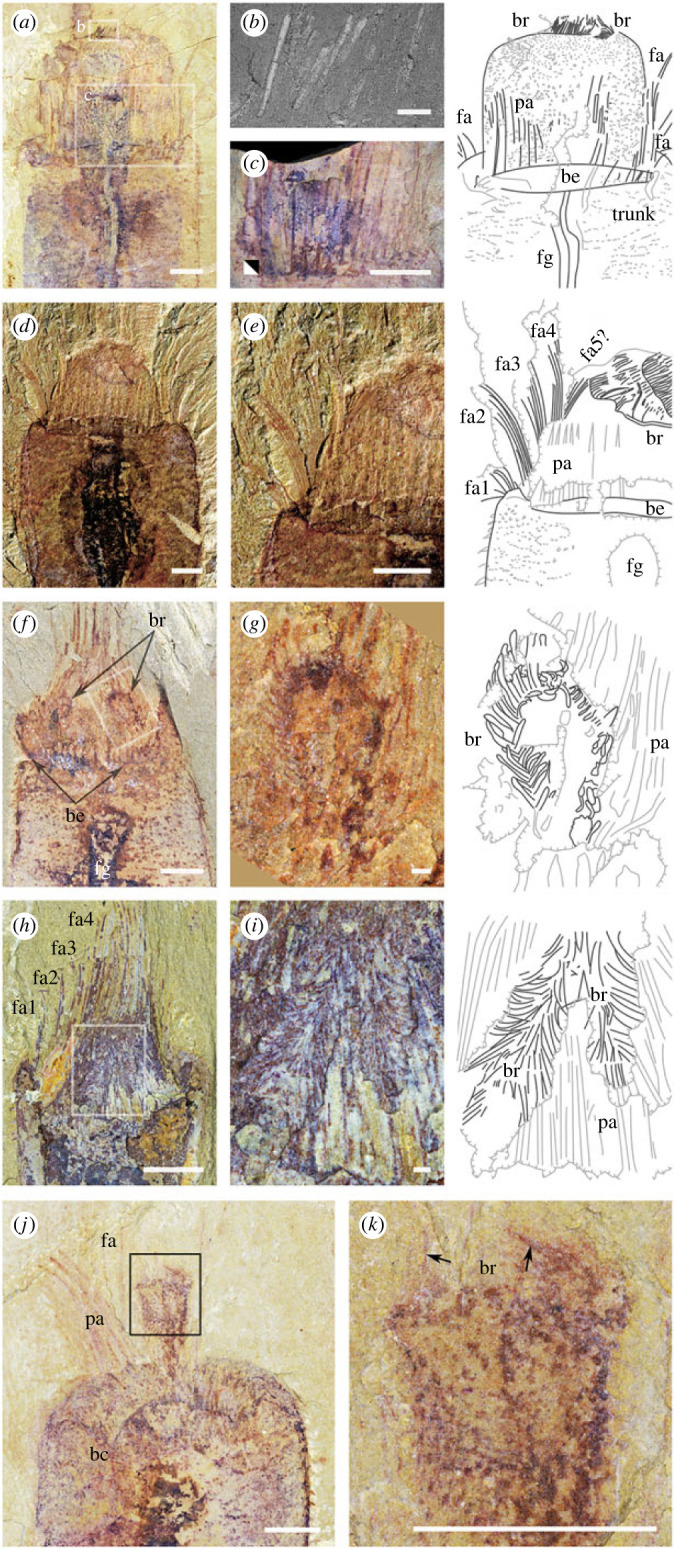
*Iotuba chengjiangensis* head. (*a–c*) ELI-S-007AB, branchiae represented in black in interpretative drawings; (*b*) backscatter scanning electron micrograph showing relief and elevated iron content of branchial filaments; (*c*) flipped image of counterpart corresponding to region boxed in (*a*) showing palisade of spines; (*d,e*) ELI-S-010, head partly retracted, flanked by palisade and at least four fascicles of spines, with distal branchiae; (*f,g*) ELI-S-003B; head partially retracted; branchiae visible beneath palisades; (*h,i*) ELI-S-011, head partially retracted, showing branchiae and fascicles of spines; (*j,k*) ELI-S-004A, partially withdrawn head showing longitudinal (left arrow) and transverse (right arrow) orientation of branchial filaments, which remain terminal even as head is withdrawn. High-resolution images at Figshare [32]. Scale bars: (*a*,*c*–*f*,*h*,*j*–*k*), 2 mm; (*b*,*g*,*i*), 200 µm. Abbreviations: be, basal element of palisade; br, branchiae; fa, fascicle of spines; fg, foregut; pa, palisade of spines.

A short head, flanked by elongate spines, can be withdrawn into the anterior trunk (figures 1 and 2). It bears two horseshoe-shaped structures, each bearing 60–100 filaments that are 300–800 µm in length and occur at regular intervals of 65–100 µm (figure 2). We interpret these paired structures as branchiae; being distinct, they cannot be interpreted as a single lophophore. The filaments may be straight (figure 2*a,b*) or curved (figure 2*g,i*), indicating an originally flexible constitution; their preservation in both two (figure 2*d–k*) and three (figure 2*b*) dimensions is typical of chemically reactive tissue in Burgess Shale-type deposits (e.g. euarthropod digestive glands [33]; nectocaridid gills [34]).

**Figure 3.**
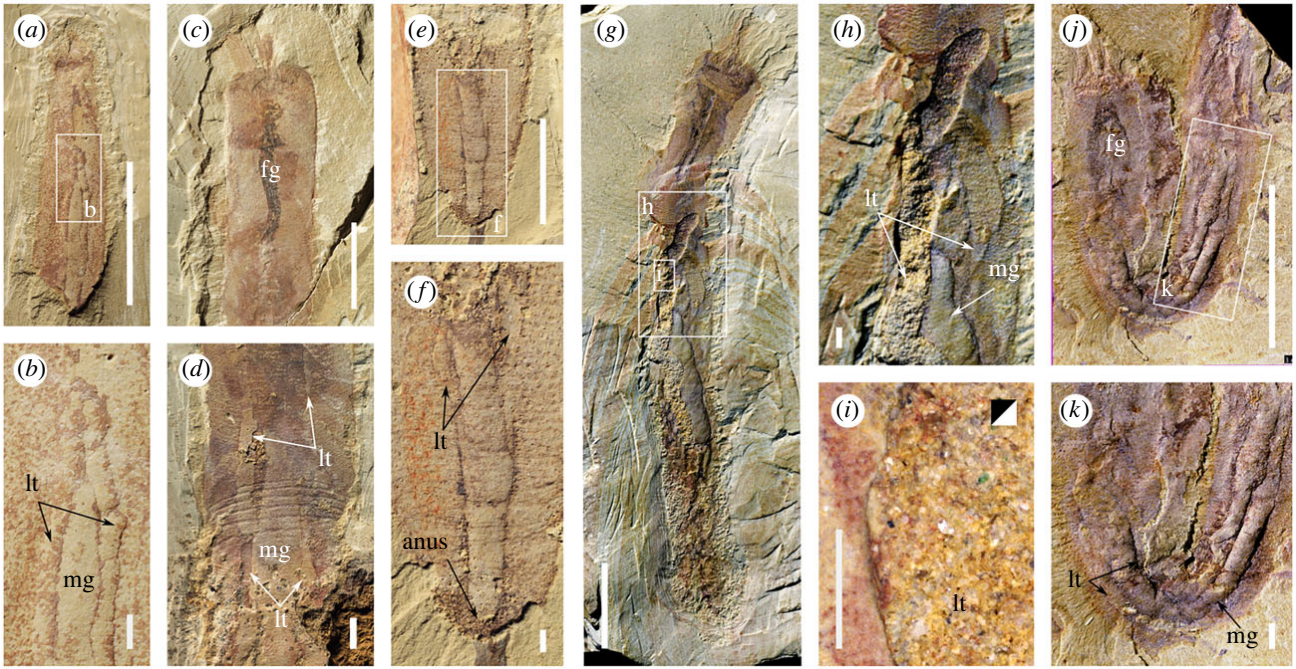
Internal anatomy of *Iotuba chengjiangensis*. (*a,b*) ELI-S-006, midgut and lateral mineral-filled tubes; (*c*) ELI-S-004A, distinct preservation of foregut; (*d*) ELI-S-007A, lateral mineral-filled tubes parallel to midgut; (*e*,*f*) ELI-S-009, posterior trunk, showing distal termination of lateral tubes; (*g–i*) ELI-S-005A; (*h*) anterior termination of lateral tubes; (*i*) (counterpart, image flipped), coarse mineral grains in gut; (*j,k*) ELI-S-008, folded specimen with bulb-shaped foregut. High-resolution images at *Figshare* [32]. Scale bars: 10 mm except enlargements (*b*,*d*,*f*,*h*–*i*,*k*,*n*), 1 mm. Abbreviations: fg, foregut; lt, lateral tube; mg, midgut.

**Figure 4.**
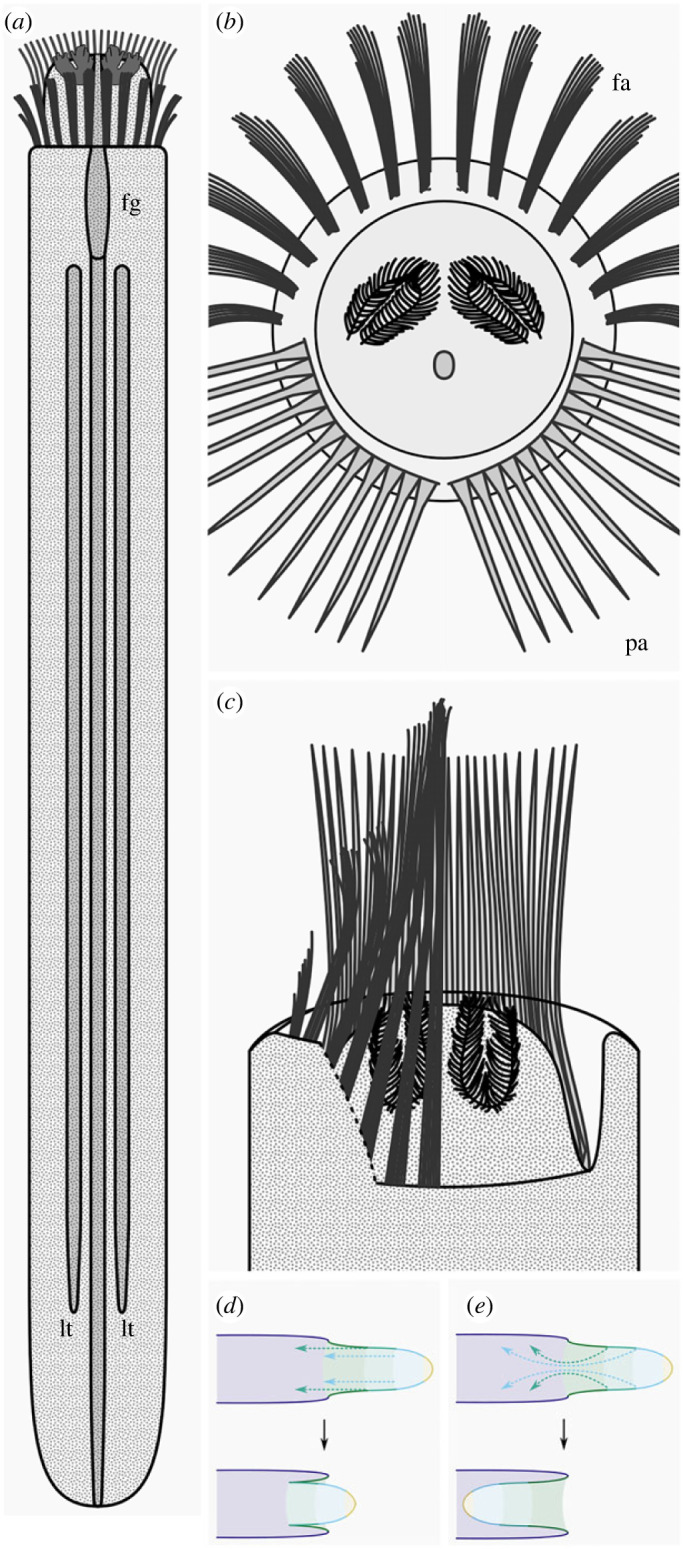
Reconstruction of *Iotuba*. (*a*) Dorsal view; (*b*) anterior view; (*c*), dorsal view, right-hand fascicles omitted to display retracted head; (*d*,*e*), schematic of a hypothetical worm showing withdrawal of an eversible head by: (*d*), retraction, as in *Iotuba*; (*e*), involution, as in ecdysozoan worms. Abbreviations: fa, fascicle of spines; fg, foregut; lt, lateral tube; pa, palisade of spines.

The base of the head is flanked by spines. Two symmetrically disposed palisades of *ca* 12 spines and a perpendicular basal element (figure 1*g,h*; 2*a,c–j*), interpreted as ventral, are complemented by a dorsal series of smaller spine fascicles (figures 1*a–c*; 2*a,d,e, h–j*). The typical spine is gently curved, with its convex surface directed centripetally, and measures 150 × 5 000 µm. Some spines are slightly bent at their distal extremity (figure 2*h*); this deformation is consistent with an originally non-mineralized composition. The fascicles and arrays of spines are splayed centripetally when the head is fully extended, but angle inwards as the head starts to retract, forming a closed cage that can be withdrawn a short distance into the body along with the body (figures 1*c,g,h*; 2*j,k*; 3*c,g,j*; 4*a,c*).

Withdrawal of the head commences with its narrowing (figures 1*a–c,h*; 2*j–k*; 3*c, g*). The consistent position of the branchiae at the anteriormost limit of the head (figure 2) indicates that the head is withdrawn by retraction without changing its shape, as a hand may be withdrawn into a sleeve (figure 4*d*). This contrasts with involution, in which the pharynx turns inside out through the mouth (figure 4*e*).

**Figure 5.**
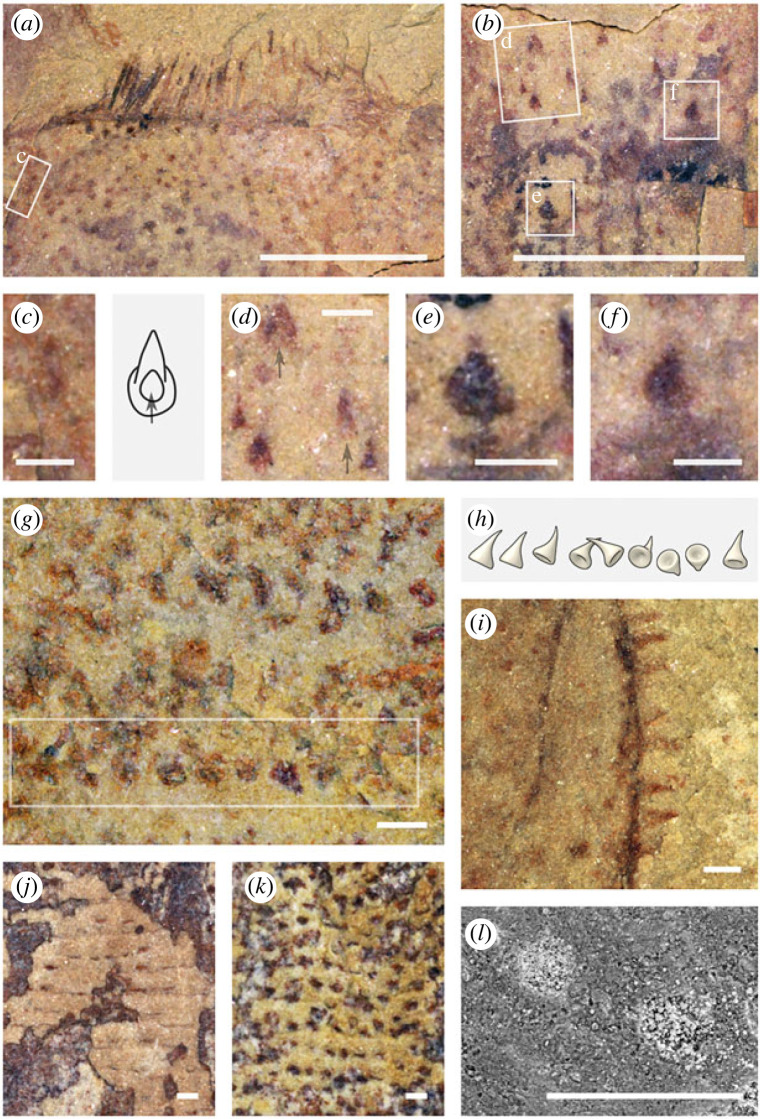
Epidermal ornament in *Iotuba chengjiangensis*. (*a*–*f*) ELI-S-007A, conical, anterior-directed papillae on head; outline of circular base prominent in (*b*,*c*,*e*,*f*); basal invagination visible in (*c*,*d*); (*g*) ELI-S-002A, detailed outline of trunk papillae; (*h*) reconstruction of original trunk papilla morphology, corresponding to boxed region in (*g*); (*i*), ELI-S-004A, outline of trunk papillae preserved on lateral margin of trunk; (*j*–*k*) ELI-S-011, impressions of papillae on inner (*j*) and outer (*k*) surfaces of trunk; (*l*), ELI-S-005A, electron micrograph showing pyrite pseudomorphs in papilla cavities. High-resolution images at *Figshare* [32]. Scale bars: 200 µm, except (*a*,*b*) (2 mm).

The surface of the head bears conical papillae with round hollow bases that extend into a distal spine with a single acute termination (figure 5*a–f*). The irregular arrangement of the papillae approximates but does not achieve close packing.

Externally, the trunk bears transverse rows of reinforced trunk papillae (figure 5*g*–*k*), spaced at around 300–350 µm. The preservation—and by implication constitution—of the papillae is similar to that of the spine arrays. The conical trunk papillae tend to be more robust than hooks on the head, indicated by their greater relief and the infilling of their internal cavity with iron minerals (originally pyrite; figure 5*l*; [32]). Each papilla has an almost hemispherical basal region that narrows distally into a pointed projection, which may be straight or gently curved (figure 5*h*). Six transverse grooves with a 1 mm spacing occur in the mid-trunk of just a single specimen (figures 1*g*, 3*d*), perhaps representing an artefact of preservation.

The digestive tract comprises an often prominent foregut preserved as a dark carbonaceous region that forms a funnel- or bulb-shaped cavity, narrowing to a straight cylindrical tube (figures 1*c–h*; 2*a,d,f,j,k*; 3). This opens into a broader, mineral-filled axial hindgut that continues to the posterior end of the trunk (figures 1*b–f*; 2*j*; 3*a,b,d–k*).

Two narrower tubes, occasionally exhibiting sausage-like constrictions (i.e. boudinaged), run parallel to the gut, starting and terminating around one body-width from each end of the trunk (figures 1*a–f*; 3). The lateral tubes are filled with coarse mineral grains that probably reflect diagenetic replication of labile tissue (per [33]). The two tubes share a common mode of preservation that is distinct from the digestive tract, which is never boudinaged, typically broader (figure 3*a–f*), and sometimes a different colour (figure 1*a–c*) or composition (figure 3*d,h*).

In three of the largest specimens, a chancelloriid is associated with the posterior trunk (figure 1*g,h*; [32]); the consistent position of the chancelloriid components relative to the trunks suggests that the superposition reflects ecology rather than taphonomy.

**Figure 6.**
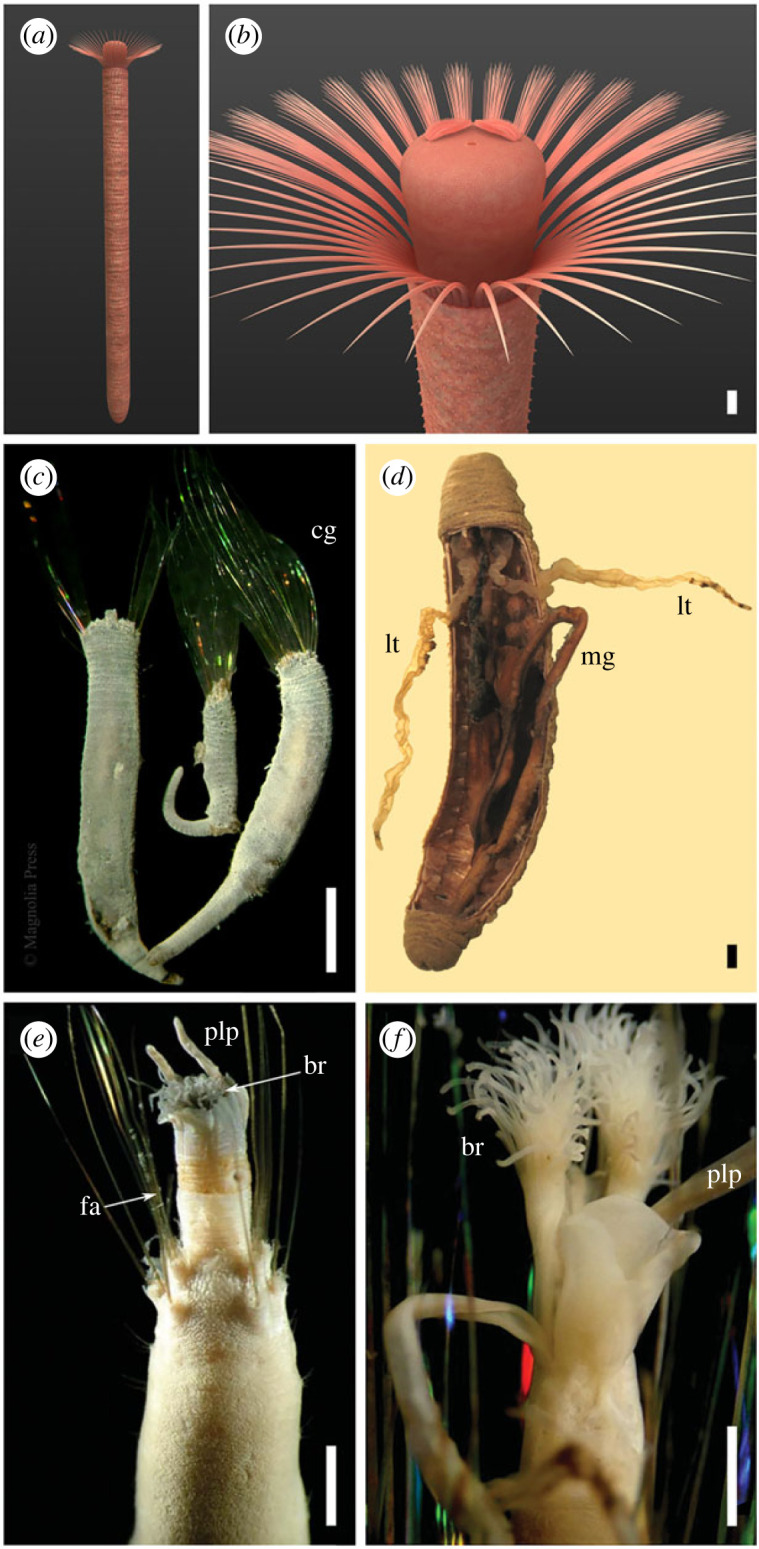
Comparison of *Iotuba* with extant flabelligerids: (*a*,*b*) life reconstruction of *Iotuba;* (*c–f*) photographs of extant flabelligerids by Sergio Salazar-Vallejo, reproduced with permission from the copyright holders (withheld from open access agreement): (*c*) *Semiodera tenera* [35], with well-displayed cephalic cages, heads partly or fully retracted; (*d*) dissection of *Brada inhabilis* [36], showing extensive nephridia; (*e*), *Stylaroides monilifer* [37], everted head showing palps and branchial filaments; (*f*) *Stylaroides hirsutus* [37], pair of fully everted branchiae. (*e*,*f*) Copyright © Unione Zoologica Italiana, reprinted by permission of Taylor & Francis Ltd, http://www.tandfonline.com on behalf of Unione Zoologica Italiana. Scale bars: 2 mm. Abbreviations: br, branchiae; cg, cephalic cage; fa, fascicle of spines; lt, lateral tube (nephridia); mg, midgut; plp, palp.

*Affinity.* Our re-evaluation of *Iotuba* (figure 6*a,b*) finds no evidence for a lophophore, stalk or U-shaped gut, the features on which a phoronid interpretation was originally founded [17,18]; as such, it is necessary to re-consider its affinity. Correct classification of Palaeozoic fossils is complicated by their antiquity: taxa in deep evolutionary positions can display unexpected combinations of derived and ancestral characteristics [38], and convergent evolution can lead to distantly related taxa exhibiting superficially similar body organizations, which can be identified as independently derived only by the comparison of specific constructional details [39].

A full appreciation of these concepts is essential to reaching correct phylogenetic conclusions. For example, the Silurian worm *Acaenoplax* [40] exhibits dorsal valves, a posterior respiratory cavity, and multiple gills. Though present in no extant mollusc, this combination of characters is inherited from the progenitor of Mollusca and thus secures a phylogenetic classification within this clade [41]. Conversely, superficially polychaete-like sclerites in *Acaenoplax* [42] can be recognized as convergent—and thus no indicator of an annelid affinity—by recognizing that their disposition is incompatible with the parapodial distribution of true polychaete chaetae [43].

To confidently reinterpret *Iotuba*, then, convergent similarities must be distinguished from authentic homologous features. Importantly, the most striking features of *Iotuba* are among the least phylogenetically instructive. A vermiform body and a semi-regular armature of cuticular sclerites characterizes cnidarian-grade organisms [44] (stem bilaterians?) as well as early representatives of many major bilaterian clades—including ecdysozoans, aculiferan molluscs [40], annelid worms [3], sipunculans [45] and brachiozoans (brachiopods and relatives) [46]. Such a morphology either characterized the ancestral bilaterian or evolved multiple times independently; either way, it does little to constrain the affinity of *Iotuba*. Likewise, an eversible anterior trunk has arisen on at least five separate occasions across Metazoa, including in gastrotrichs, acanthocephalans, ecdysozoans, sipunculans and annelids. A compelling designation of *Iotuba* to one of these groups requires much more basis than the shared presence of a feature that has evolved so many times independently.

Do any of these proboscis-everting groups exhibit the detailed constructional similarities necessary to substantiate an affinity with *Iotuba*? Not gastrotrichs, which typically have differentiated dorsal and ventral surfaces and a differentiated trunk, with cataphract trunk armature, and an unarmoured head surrounded by sensory cilia, not spines. Within Ecdysozoa, Cambrian ‘archaeopriapulids’ seem at first blush to offer a promising point of comparison [47,48]. Outwith the derived clade Panarthropoda, Cambrian ecdysozoans exhibit a conserved body plan. All *bona fide* representatives exhibit a specific and distinctive anterior organization that can be recognized on morphological grounds [14,49]: the anterior trunk is differentiated into an eversible introvert armoured with radially arranged rows of hooks or spines; the mouth is surrounded by a radially symmetric ring of spines; the proximal region of the eversible pharynx is unarmoured; and the distal pharynx is arrayed with one or more regions of quincuncially arranged teeth in which spines emerge as extensions of a raised subtriangular arch housed on a polygonal basal pad or spur [49].

Despite a superficial similarity, however, the anterior region of *Iotuba* does not conform to any aspect of this pattern. The anterior trunk is not eversible; it is not differentiated, either morphologically or by its armature; the junction between its trunk and eversible head is adorned not with the radial array of elements that is retained even within Panarthropoda [50], but with a bilateral arrangement of spines, including spines in fascicles; the *Iotuba* head does not bear the unarmoured region that characterizes the proximal ecdysozoan pharynx; and the head papillae are neither arranged nor constructed in the fashion of ecdysozoan pharyngeal teeth.

The absence of detailed anatomical correspondence between ecdysozoans and any aspect of *Iotuba* militates strongly against a close phylogenetic relationship. A position within the ecdysozoan crown group would imply that the anterior morphology of *Iotuba* was modified to a degree unparalleled anywhere in Ecdysozoa, whereas the incorporation of ‘branchiae’ into the pharynx is particularly difficult to reconcile with an ecdysozoan body plan.

Sipunculans are perhaps more promising: certain living and fossil sipunculans [45,51] exhibit conical trunk papillae, large, paired, axis-parallel nephridia, and—in their perioral tentacles—a potential equivalent to the (admittedly non-perioral) *Iotuba* branchiae. Under this model, the *Iotuba* head would correspond to the sipunculan introvert, whose hooks can resemble those of the *Iotuba* trunk [52]. This comparison is inexact: the sipunculan introvert is withdrawn by involution, rather than retraction; has a much higher length : width ratio; and is differentiated, with the tentacles occupying a distinct, unarmoured and articulated region of the distal introvert, the cephalic collar, which has no obvious equivalent in *Iotuba*. If these constructional differences are overlooked, then *Iotuba* might conceivably be accommodated in the sipunculan stem lineage at a point before the anus migrated to the anteriormost trunk or introvert, though the fascicles and palisades of spines must be derived by some *ad hoc* pathway from a presumed annelid ancestor.

Flabelligerid annelids (cage worms) (figure 6*c–f*) offer a more compelling point of comparison. These worms are characterized by a retractable, faintly papillate head, flanked or encircled by a ‘cephalic cage’ made up of fascicles of elongate spines (chaetae). The configuration of this cage ranges from distinct fascicles of chaetae with clear vestiges of a segmental arrangement (e.g. figure 6*e*), recalling the fascicles of chaetae in *Iotuba* (though the nature of fossil preservation precludes the identification or differentiation of individual segments) to the single-layer, broadly radial palisades in *Flabelliderma* and *Flabelligera* [36], which recall the *Iotuba* palisades. The flabelligerid head exhibits horseshoe-shaped branchial plates with numerous filamentous projections [36] (figure 6*e,f*). Flabelligerid nephridia can form long, subcylindrical, axis-parallel structures of a similar width to the intestine (figure 6*d*) that offer a likely interpretation for the lateral tubes. To complete the picture, a retractile anterior end, cylindrical body, prominent trunk papillae (sometimes in transverse rows, albeit lacking sclerotization; figure 6*c,e*) and a lack of prominent annulation (figure 6*e*) characterize flabelligerids and many other cirratuliforms [36].

Flabelligerids, then, are the only animal group to exhibit plausible homologues of each major organ system within *Iotuba*. Of course, given the great age of the fossil, it is not expected that these structures will be identical to representatives of the crown group—just as the forelimbs of dinosaurs (stem-group birds) are homologous to, but morphologically different from, the wings of crown-group birds. In Cambrian taxa, homologous features may not yet exhibit the full set of properties that characterize their manifestation within the crown group, which has been winnowed by extinction to represent a subset of the morphological diversity present early in a clade's history [53]. Indeed, *Iotuba* differs from most crown-group flabelligerids in the robust cuticularization of its papillae—though precedents for the independent sclerotization of cuticle can be readily found in many metazoan groups (e.g. [52,54–57]). The internal elements perpendicular to the base of spine palisades do not have an exact parallel in flabelligerids, though the internal rod-like skeletal chaetae (aciculae) of other polychaetes offer a plausible analogue. Even if the sturdiness of the cephalic cage and the precise arrangement of branchial filaments is not replicated by any individual flabelligerid species, the great diversity of arrangements within the family [36] demonstrates the range of form that can evolve from homologous structures.

Conversely, because Cambrian fossils tend to occupy deep phylogenetic positions, they seldom possess all the features of extant relatives [38]. The absence of a mucoid tunic, found in most extant flabelligerids, doubtless reflects the negligible fossilization potential of mucus. Despite being a key element of the cirratuliform body plan [58], palps have been lost in the extant flabelligerid *Buskiella* [59] and were presumably lost independently in *Iotuba*. A secondary loss of trunk chaetae in *Iotuba* would be surprising, but not without precedent; the diminutive nature and even absence of trunk chaetae in certain extant flabelligerids [25] arguably suggests a diminished functional role and a reduced selective pressure for their retention.

Taken together, we acknowledge that the case for an annelid affinity requires *Iotuba* to exhibit a somewhat derived morphology relative to the inferred ancestral state of flabelligerids, and that the correspondence with structures in extant annelids is imperfect. However, we consider it more parsimonious to treat the features observed in *Iotuba* as potential homologues of organs that occur in combination in a known group than to treat each feature as an independent innovation that is unique to *Iotuba*.

The alternative to a flabelligerid position is extreme convergence from a potentially non-annelidan progenitor. Such a proposal is difficult to falsify—yet we have been unable to concoct a compelling scenario. There is no obvious home for *Iotuba* among the dorsoventrally differentiated cataphract metazoans that populate the stem lineages of the lophotrochozoan phyla [60–63]. Of the myriad worm-like taxa with similar overall dimensions (e.g. [14,64,65]), perhaps the most relevant is *Acosmia maotiania* [66], a Chengjiang fossil with lateral tubes alongside its gut (see figs 1 and 3 of [66]). However, *Iotuba* and *Acosmia* differ in almost every morphological detail. *Iotuba* has an undifferentiated trunk; the *Acosmia* trunk is differentiated into an introvert with posterior-directed spinose elements; a regularly annulated mid-trunk with no discernible sclerites; and a posterior region with plate-like sclerites. These sclerites comprise a central boss and a circumferential groove, making them more similar to palaeoscolecid plates [67] than the spinose, basally indented papillae of *Iotuba*. *Iotuba* has an eversible head; *Acosmia* lacks a head, and its pharynx is permanently retracted. *Iotuba* has an unarmoured foregut; the *Acosmia* pharynx contains sclerotized internal elements, reminiscent of the triradial, stylet-bearing pharynx of nematodes. *Acosmia* lacks any parallel to the branchiae, or anterior spines of *Iotuba*; *Iotuba* has no equivalent to the raised external ‘lip’ of *Acosmia*. Taken together, a close affiliation is undermined by the different organization of the gut and trunk, and does not recast the numerous autapomorphies of each taxon in a framework of homology by common descent.

In any case, *Acosmia* itself lacks a secure phylogenetic placement. Its interpretation as a total-group ecdysozoan [66] rests on its terminal mouth and annulated vermiform body—a non-specific suite of characters that also characterizes, for example, many polychaetes. *Acosmia* is a rogue taxon in ecdysozoan phylogenies: it may sit in the nematoid crown group, the ecdysozoan stem, or elsewhere [68]. This inconsistent phylogenetic position shows that *Acosmia* does not fit neatly into the current understanding of ecdysozoan evolution. The underwhelming and ambiguous evidence for the affinity of *Acosmia* means that its potential affiliation with *Iotuba*, even if substantiated, would do little to ground either taxon in a phylogenetic framework.

**Figure 7.**
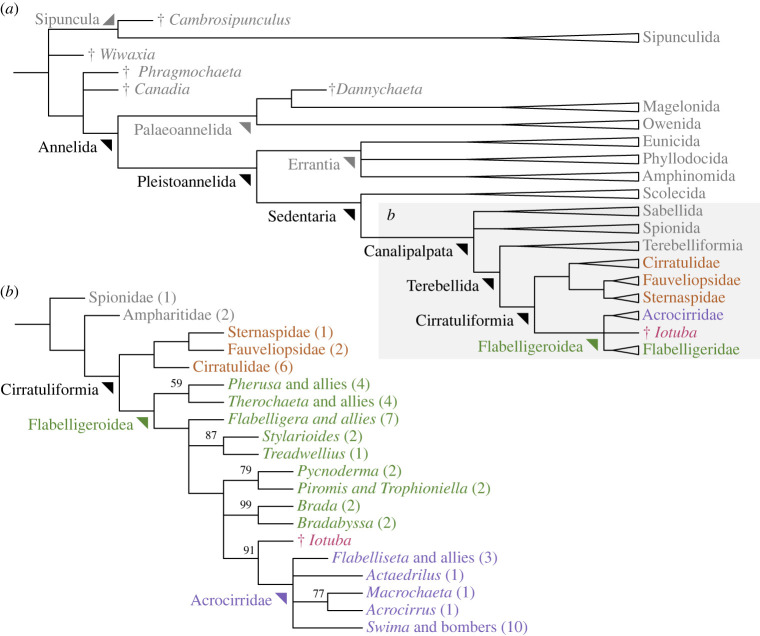
Phylogenetic position of *Iotuba*: (*a*) outline phylogeny of Annelida, showing representative Cambrian fossils (marked with a dagger †); box marks scope of detailed phylogenetic analysis; (*b*) consensus of Bayesian, maximum likelihood and parsimony topologies, showing derived position of *Iotuba* within paraphyletic ‘Flabelligeridae’; parentheses denote number of taxa within clade; node labels, Bayesian posterior probabilities (where less than 100%).

An alternative test of convergent evolution can be provided by phylogenetic analysis. To evaluate the case for homology between the *Iotuba* and flabelligerid body plans, we incorporated *Iotuba* in a new phylogenetic dataset comprising morphological and molecular data from cirratuliform annelids (electronic supplementary material). Bayesian inference, maximum likelihood and inapplicable-corrected parsimony (under equal and implied step weighting) all identified *Iotuba* as a crown-group flabelligeroid, nested within a paraphyletic Flabelligeridae as sister to Acrocirridae (figure 7). Characters uniting *Iotuba* with Acrocirridae include the loss of a caruncle and cephalic hood (though these are convergently re-gained in certain acrocirrids). If Flabelligeridae is constrained to be monophyletic, *Iotuba* plots as its sister taxon, remaining within the Flabelligeroidea crown group. As such, whatever the relationships between the cirriform families, interpreting *Iotuba* as a total-group flabelligerid is parsimonious and is consistent with morphological and molecular data.

## 3. Discussion

Annelid worms are rare in Chengjiang [69,70]. *Ipoliknus* [70] bears ‘sclerites’ that resemble the robust, cuticularized papillae of *Iotuba*, so conceivably also belongs to the papilla-bearing subclade of Cirratuliformia [24]—though available material is inadequate to substantiate this hypothesis. Detailed comparison with the undescribed ‘New Taxon 1’ [32,66] or *Dakorhachis* [65], whose segmented trunk and cage of terminal spines are somewhat reminiscent of *Iotuba*, is precluded by the limited preservation of available material.

Previously known annelids from the early Cambrian belong either to the annelid stem group [2], or to the early-diverging lineage Palaeoannelida [5]. As such, there has been no evidence of a diversified annelid crown group until the Ordovician, when machaeridian phyllodocids [71] and eunicid jaw elements [72] document the divergence of the pleistoannelid subclass Errantia, to which all known Ordovician–Devonian annelids belong [73], from its probable sister clade Sedentaria [74], which includes the flabelligeroids.

Membership of Flabelligeroidea would grant *Iotuba* a derived position within Annelida, placing it within the crown group of the nested clades Cirratuliformia, Sedentaria and Pleistoannelida (figure 7). This would draw back the scant fossil record of Sedentaria by 200 Myr, close to the first fossil evidence of annelids [75].

Because Flabelligeroidea is deeply nested, each of its parent clades necessarily diverged before it originated—which would imply that the annelid crown group was already highly diverse by Chengjiang time. The non-preservation of this diversity represents a paradox [53], but could be resolved if early annelids preferred environments that precluded exceptional preservation: stem-group annelids, at least, display a preference for particular environmental conditions [76,77].

From a wider perspective, the cryptic Cambrian roots of annelid diversity point to an earlier radiation than previously expected [2]: a flabelligeroid interpretation of *Iotuba* would pull the divergence of the major pleistoannelid lineages back into the contracted period of evolutionary innovation that marked the opening of the Phanerozoic eon.

## 4. Methods

As the combined analysis of morphological and molecular data increases the concordance between reconstructed and independently well-corroborated trees [78–80], we constructed a new dataset of morphology + mitochondrial DNA for 60 extant annelids and *Iotuba*.

Our morphological data comprise 82 discrete characters. Some character formulations were sourced from previous morphological datasets [24,25] and reformulated to follow best practices for character construction [81–83]. Characters were scored for each taxon based on the most recent available literature, resulting in the revision of many codings from previous datasets. The morphological dataset was then reduced by safe taxonomic reduction.

Mitochondrial DNA sequences for the 16S, 18S, 28S, cytochrome *b* and cytochrome *c* oxidase I loci were obtained by searching GenBank by locus and taxon, and using a BLAST search [84], with sequences listed by [24,85–87] used to seed searches.

Sequences were aligned using SATe 2.2.7 [88–91] following an established protocol [92]: alignment was conducted using MAFFT [93] with the Opal merger [94] and the FastTree tree estimator [95], using the generalized time-reversible model [96] with 20 gamma-distributed rate categories and the SATe-II-ML settings, stopping 15 iterations after the last improvement to alignment score. Raw and aligned sequences are available in the electronic supplementary material.

Phylogenetic analysis was conducted using maximum likelihood, Bayesian inference and maximum parsimony. Model and partition selection for probabilistic analysis was conducted using ‘ModelFinder’ [97,98]; optimal partitioning and models are listed in the electronic supplementary material. Maximum-likelihood tree search was conducted in ‘IQ-Tree’ [99], allowing each partition to have its own evolutionary rate over common branch lengths [98]. Bayesian analysis was performed in MrBayes 3.2.7a [100] using Bayesian mixed models, and the models identified by ModelFinder. Four runs of eight chains were run for 5 million generations, sampling every 500 generations and discarding the first 10% of samples as burn-in. Convergence was indicated by potential scale reduction factor = 1.00 and an estimated sample size of greater than 200 for each parameter. Parsimony search was conducted using Fitch parsimony [101] in ‘TNT’ [102] (supported by the Willi Hennig Society), and with a correction for inapplicable morphological tokens [82] in the ‘R’ package ‘TreeSearch’ [103–105]. We employed the parsimony ratchet [106] with implied weights [107], using concavity constants of 3, 4.5, 7, 10.5, 16, 24, 36, 54 and ∞ (i.e. equal step weights). Summary trees are presented with node support values calculated after removing rogue taxa [108].

A subsample of the individual bifurcating trees reconstructed by each approach was compared by mapping the clustering information and quartet distances [109,110] between each pair of trees into two dimensions using classical multidimensional scaling [111], using the R packages ‘Quartet’ and ‘TreeDist’ [112,113]. Adequacy of projection was indicated by trustworthiness and continuity metrics above 0.90 and minimal deformation of the minimum spanning tree [114–117]. This analysis indicated that all methods except uncorrected Fitch parsimony converged onto a similar region of tree space. Full results are provided in the electronic supplementary material.

## Data Availability

All data are available in the main text, or the electronic supplementary material [118]. High-resolution images of specimens accessioned at the Early Life Institute are available from *Figshare*: https://doi.org/10.6084/m9.figshare.c.4204718 [32].
